# The Influence of Initial Surface Roughness on the Current-Carrying Friction Process of Elastic Pairs

**DOI:** 10.3390/ma18020370

**Published:** 2025-01-15

**Authors:** Zhenghai Yang, Wenbo Li, Xiaomeng Zheng, Mengfeng Zhao, Yongzhen Zhang

**Affiliations:** 1 School of Materials Science and Engineering, Henan University of Science and Technology, Luoyang 471023, China; lwb00818@163.com (W.L.); 13167771852@163.com (M.Z.); 2 National United Engineering Laboratory for Advanced Bearing Tribology, Henan University of Science and Technology, Luoyang 471023, China; 9906256@haust.edu.cn (X.Z.); yzzhang@mail.haust.edu.cn (Y.Z.)

**Keywords:** current-carrying friction, connector, surface roughness, furrowing, arc erosion

## Abstract

To investigate the effect of the initial surface roughness on the performance at the initial stage of the current-carrying friction of an elastic friction pair, experiments were conducted using a self-made current-carrying friction and wear tester. The results indicate that under the experimental conditions, the lifespan of the friction pair decreases as the surface roughness and load decrease. When the surface roughness is Ra 0.2 μm and the load is 0.025 N, the lifespan is the longest, reaching 320 cycles, with an average contact resistance of 0.045 Ω and a standard deviation of 0.009 Ω. During the normal service period of the friction pair, the main wear mechanism is furrowing. As adhesion and tearing occur, the electrical contact performance begins to degrade. The impact of arc erosion on the wear surface is far greater than that of mechanical wear. This provides a reference for the design and manufacture of current-carrying friction pairs represented by connectors.

## 1. Introduction

Under hot-swapping conditions, electrical connectors are typical elastic contact current-carrying friction pairs [1,2,3]. The mechanical lifespan of connectors is typically only dozens to thousands of cycles [4]. They are often made of plated materials (of a micron-scale thickness) [5] and are used directly after manufacturing. Classical friction theory relies on running-in [6], which does not consider the initial state of the friction surfaces, making it unsuitable for the service process of connectors. With advancements in technology, the performance requirements for electrical connectors under hot-swapping conditions have become increasingly stringent [7,8,9], and traditional experience can no longer address them. Therefore, it is urgent to study the effects of the surface conditions on connector performance.

Previous studies have extensively investigated the factors affecting the performance of connectors, such as the material, insertion/extraction speed, current, temperature, and humidity. Werner Österle et al. [10] prepared electroless nickel–phosphorus coatings, comparing them with nanocrystalline nickel coatings, and found that nickel–phosphorus coatings exhibited better anti-cold welding and tribological properties. Jing Ni et al. [11] studied the evolution of the electrical connector contact performance at different speeds and found that an increased insertion/extraction speed significantly accelerated the wear. Wenxin He et al. [12] used a numerical simulation to investigate the impact of the current intensity on wear and electrical contact performance, revealing that the wear depth first increased and then decreased as the current increased. L. Ke et al. [13] analyzed the intermittent fault characteristics of electrical connectors under different temperature stress levels, finding that higher temperature change rates led to more frequent intermittent faults. I.H. Sung et al. [14] studied the effects of humidity on the fretting instability of the contact resistance under different sliding distances and found that increased humidity significantly stabilized the contact resistance at shorter sliding distances. Guo Fengyi et al. [15] studied the effect of surface roughness on the current-carrying performance and found that the contact resistance exhibits a “V”-shaped variation with increasing roughness. The current-carrying efficiency first increases and then decreases, the stability of the current-carrying performance gradually deteriorates, and the friction coefficient gradually increases. These studies either did not focus on the impact of roughness on performance or did not apply it to the current-carrying friction stage corresponding to the mechanical lifespan of connectors.

Du Jiebin et al. [16] prepared Ag coatings using additive manufacturing. They found that the surface roughness of the coating significantly affects the current-carrying friction performance during the running-in phase. Wang Dongwei et al. [17], in studying the effect of a normal load on the current-carrying friction behavior of graphene coatings, pointed out that the surface roughness significantly affects the performance of friction pairs in the initial stage. These studies all observed the effect of the surface roughness on the current-carrying friction performance but did not conduct in-depth investigations.

Therefore, this paper simulates the plug-in process of electrical connectors under actual hot-plugging conditions using a wire–plate friction pair, designs plate specimens with different surface roughness values, and studies the impact of the initial surface roughness on the current-carrying friction process for connectors.

## 2. Materials and Methods

**Figure 1:**
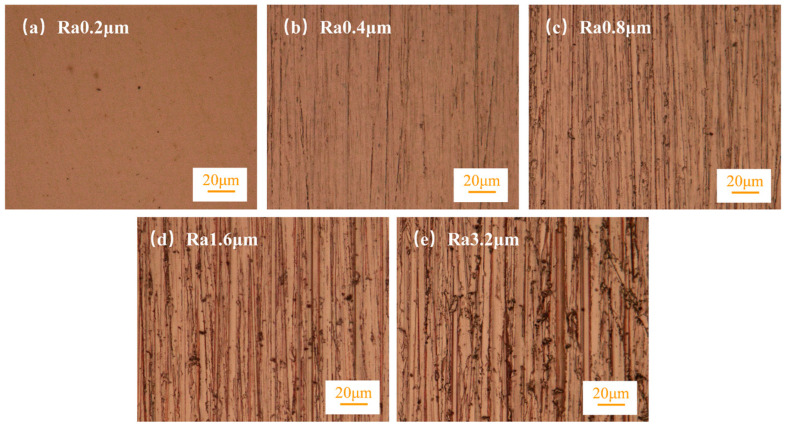
Metallographic image of plate specimen.

The experiment used a wire-to-plate elastic friction pair, where the wire specimen was commercially available brass wire (H62, Luoyang Copper Processing Group Co., Ltd., Luoyang, China) with a diameter of Φ0.4 mm. The wire was bent at a 60° angle, with the arc apex perpendicular to the plate specimen and making contact with the plate at a 2.5 mm radius arc. The plate specimen was a commercially available copper plate (T2, Luoyang Copper Processing Group Co., Ltd.). Before the experiment, the surface was polished to Ra 0.2 μm using a grinding and polishing machine (MOPAO4S, Laizhou Veiyee Testing Equipment Manufacturing Co., Ltd., Laizhou, China). Then, W3.5 to W7 metallographic sandpapers were used to grind different specimens in the same direction to obtain plate specimens with surface roughness values of Ra 0.2 μm (measured as Ra 0.194 μm), Ra 0.4 μm (measured as Ra 0.392 μm), Ra 0.8 μm (measured as Ra 0.790 μm), Ra 1.6 μm (measured as Ra 1.596 μm), and Ra 3.2 μm (measured as Ra 3.094 μm) (Figure 1) [18].

**Figure 2:**
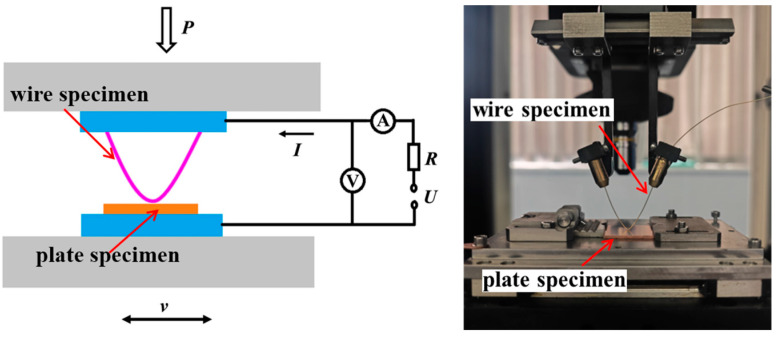
Experimental schematic diagram.

The experiment was conducted on a custom-built current-carrying friction and wear tester (Figure 2) [19]. A DC constant current source was used as the power supply. The direction of friction was perpendicular to the grinding direction of the plate specimen. The friction pair performed reciprocating sliding with a stroke of 5 mm, a frequency of 1 Hz, a current of 3 A, and a duration of 500 s (500 cycles) under loads of 0.025 N, 0.05 N, 0.075 N, and 0.1 N. After the friction and wear test, a JSM-5610LV scanning electron microscope (SEM, JEOL Ltd., Tokyo, Japan) was used to observe the worn surface, and an Rtec MFT-5000L 3D profilometer (Rtec, San Jose, CA, USA) was used to examine the 3D morphology of the wear surface. The surface elements of the material after wear were analyzed using an energy-dispersive spectrometer (EDS, JEOL Ltd., Tokyo, Japan).

## 3. Results

### 3.1. The Effect of the Initial Surface Roughness on the Frictional Performance Under Current-Carrying Conditions

**Figure 3:**
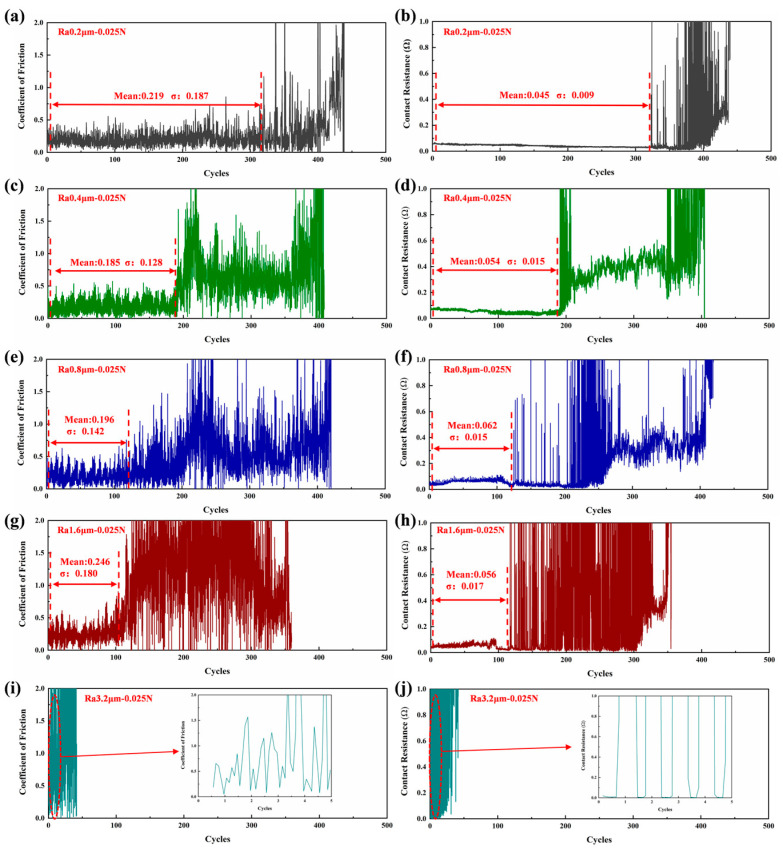
Dynamic friction coefficient and contact resistance.

Figure 3a,c,e,g,i present the dynamic friction coefficients for different initial surface roughness levels. As shown in the figure, the friction coefficient is relatively stable, with small fluctuations in the early stage of friction, with the average value ranging between 0.185 and 0.246 and the standard deviation ranging between 0.128 and 0.187. Subsequently, the average friction coefficient starts to increase sharply, with intensified fluctuations, and eventually, the test terminates with severe fluctuations in the friction coefficient. As the initial surface roughness increases, the fluctuation in the friction coefficient intensifies, and severe fluctuations occur earlier.

Figure 3b,d,f,h,j show the dynamic contact resistance for different initial surface roughness levels. From the figure, it can be observed that the contact resistance follows the same trend as the friction coefficient. In the early friction stage, the contact resistance remains stable, with small fluctuations, with an average value ranging between 0.045 and 0.062 Ω and a standard deviation between 0.009 and 0.017 Ω. Subsequently, the contact resistance fluctuates violently, with significant abrupt changes, and the electrical contact performance starts to deteriorate. At this point, the friction pair is considered to have failed, losing its good conductivity. The lifespan of the friction pair decreases with an increasing initial surface roughness, reaching a maximum of 320 cycles at Ra 0.2 μm and a minimum of 0.7 cycles at Ra 3.2 μm.

**Figure 4:**
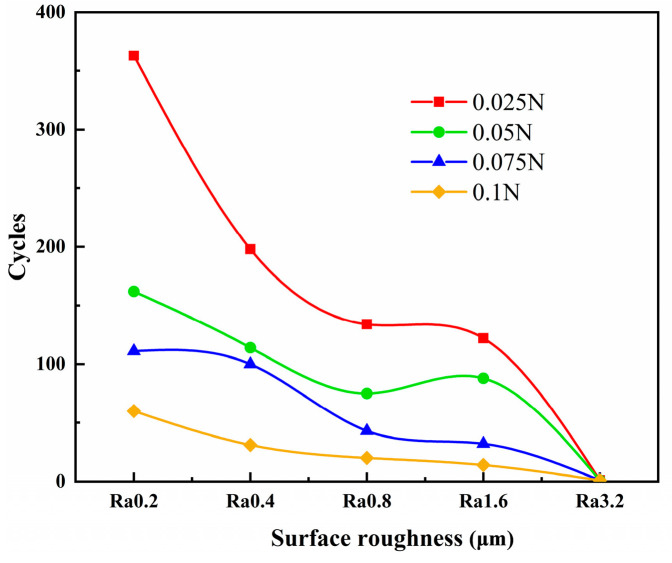
Trend chart for friction pair lifespan.

Figure 4 shows the trend in the friction pair’s lifespan varying with the surface roughness under different loads. It can be seen from the figure that the lifespan of the friction pair decreases gradually as the surface roughness increases. As the load increases, the lifespan of the friction pair gradually decreases. When the initial surface roughness is Ra 0.2 μm and the load is 0.025 N, the lifespan of the friction pair reaches a maximum of 320 cycles.

### 3.2. Damage Behavior Under Different Conditions

#### 3.2.1. Current-Carrying Frictional Behavior of Smooth Initial Surfaces

**Figure 5:**
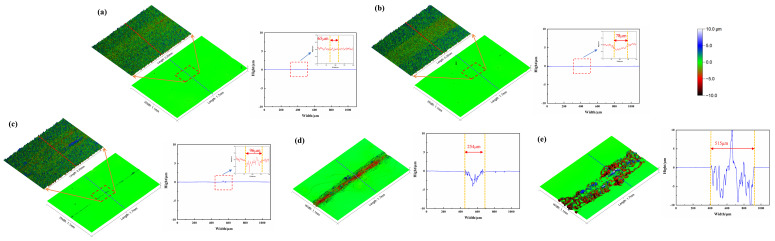
Three-dimensional morphology and height curve of the evolution process for the smooth initial surface.

Figure 5 shows the three-dimensional morphology and corresponding height curves in different cycle periods under the conditions of Ra 0.2 μm and a load of 0.025 N. The cycles are 100, 200, 300, 400, and 439, respectively. From the figure, it can be observed that after 100 (Figure 5a) and 200 (Figure 5b) cycles, the wear surface’s width is approximately 65~75 μm, and the wear primarily manifests as micro-convex peaks being flattened, gradually forming a relatively smooth wear surface. When 200 cycles are reached, the wear area’s depth is approximately 0.3~0.5 μm. After 300 cycles (Figure 5c), the wear surface’s width reaches 96 μm, and grooves of an approximately 1 μm depth appear in the wear area. From the height curve, it can be seen that the fluctuations in the wear area’s height increase. After 400 cycles (Figure 5d), the wear surface’s width sharply increases to 254 μm, and its depth is approximately 5 μm. After 439 cycles (friction pair failure, Figure 5e), the wear surface’s width sharply increases to 515 μm, and the range of fluctuations in the height curve in the wear area is between −10 μm and 10 μm.

**Figure 6:**
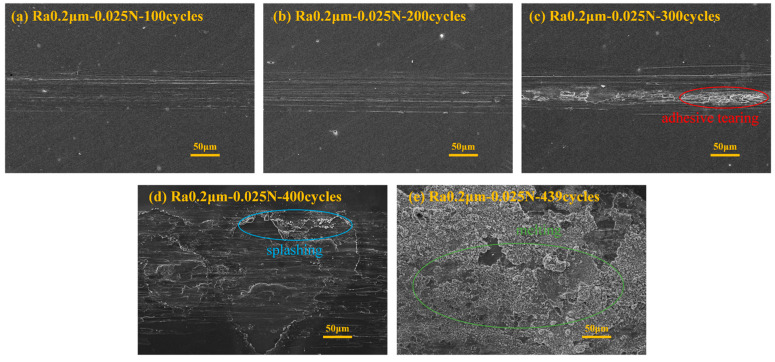
SEM image of the corresponding experiment in Figure 5.

Figure 6 presents the SEM images under the conditions corresponding to Figure 5. From the figure, it can be observed that after 100 (Figure 6a) and 200 (Figure 6b) cycles, the wear form is primarily furrowing. After 300 cycles (Figure 6c), the wear is still dominated by mechanical wear, and in addition to furrowing, adhesive wear and tearing also appear [20]. After 400 cycles (Figure 6d), in addition to mechanical wear, primarily characterized by adhesion and tearing, traces of arc erosion also appear [21]. After 439 cycles (friction pair failure, Figure 6e), the entire wear surface is almost entirely occupied by traces of arc erosion, including melting and spattering.

#### 3.2.2. Current-Carrying Frictional Behavior of Rough Initial Surfaces

**Figure 7:**
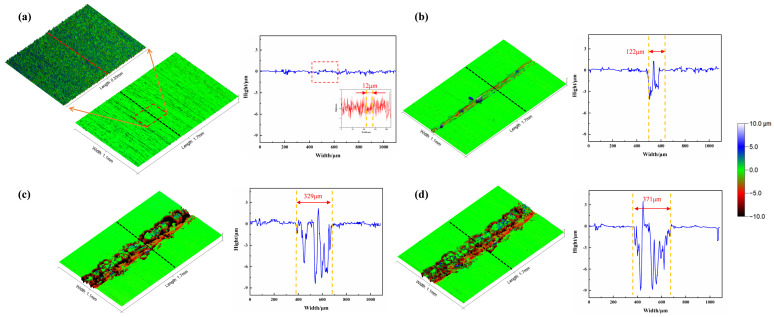
Three-dimensional morphology and height curve of rough initial surface evolution process.

Figure 7 shows the three-dimensional morphology and corresponding height curves in different cycle periods under the conditions of Ra1.6 μm and a load of 0.025 N. The cycles are 100, 200, 300, and 371 cycles, respectively. From the figure, it can be observed that after 100 cycles (Figure 7a), the wear surface’s width is approximately 12 μm. The height of the micro-convex peaks in the wear area decreases, and the valleys become shallower. The fluctuation in the height curve of the worn surface is smaller than that in the original surface. After 200 cycles (Figure 7b), the wear track’s width sharply increases to 122 μm. The wear surface shows a depth of approximately 3~4 μm, with spot-like protrusions appearing. After 300 cycles (Figure 7c), the wear track’s width sharply increases to 329 μm, and the wear surface’s depth is approximately 8~9 μm. After 371 cycles (friction pair failure, Figure 7d), the wear surface’s width reaches 371 μm, and the range of fluctuations in the wear track’s height is between −9 μm and 3 μm.

**Figure 8:**
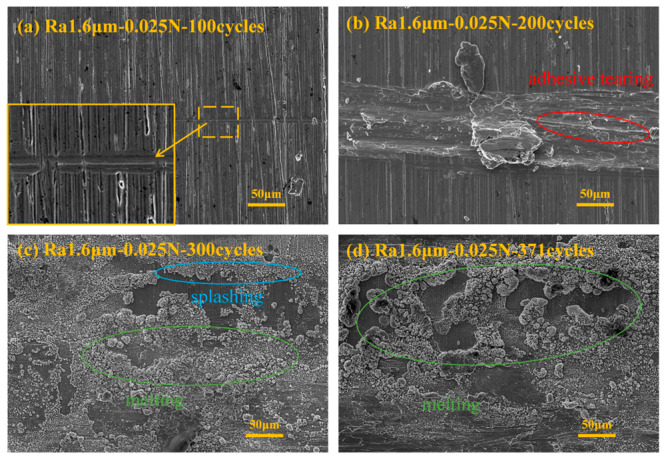
SEM image of the corresponding experiment in Figure 7.

Figure 8 presents the SEM images under the conditions corresponding to Figure 7. From the figure, it can be observed that after 100 cycles (Figure 8a), the wear form is primarily furrowing, a type of mechanical wear. After 200 cycles (Figure 8b), the wear is still dominated by mechanical wear, primarily manifesting as adhesion, tearing, and furrowing, with large accumulations of wear debris on the worn surface. After 300 cycles (Figure 8c), many arc erosion traces, such as melting and spattering, appear on the wear surface. After 371 cycles (friction pair failure, Figure 8d), the wear surface still shows severe arc erosion traces, which are more serious compared to those at 300 cycles.

#### 3.2.3. Trend in Wear Height Variations

**Figure 9:**
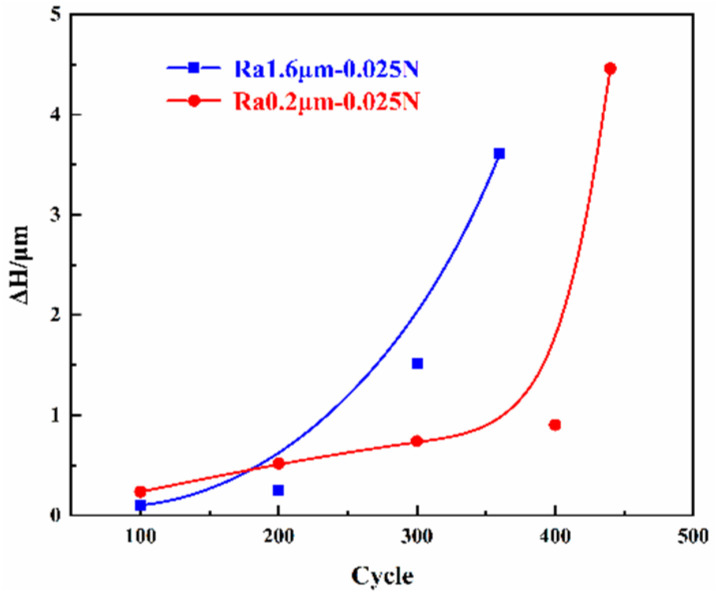
Wear height variation.

Figure 9 shows the trend in the average wear height (the absolute value of the difference between the wear surface and the original surface’s average height) as a function of the number of cycles under the conditions corresponding to Figure 5 and Figure 7. From the figure, it can be seen that as the number of cycles increases, the wear height first increases slowly and then increases sharply. In the early stage of wear, the variation in the height of the smooth initial surface is slightly larger than that in the rough initial surface. As the lifespan of the friction pair with a rough initial surface ends earlier, the variation in the height of the rough initial surface quickly surpasses that for the smooth initial surface until the tribological pair fails. The change in wear form has a significant impact on the wear height, with the appearance of arc erosion intensifying the wear.

#### 3.2.4. Effect of Arc Erosion on the Surface

**Figure 10:**
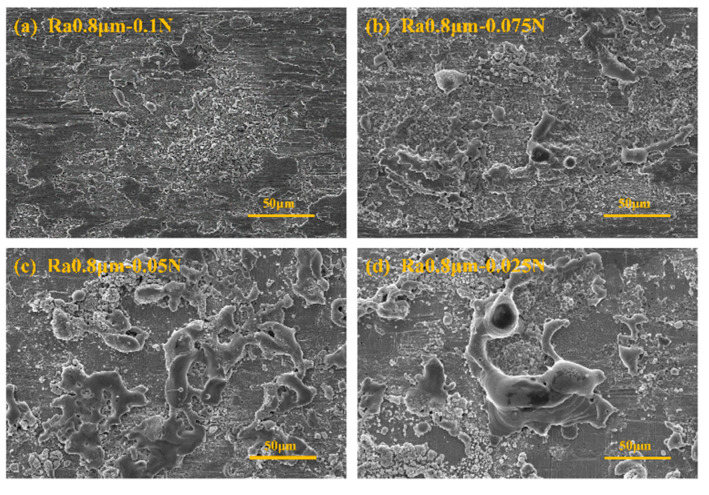
SEM image of strong electric arc erosion.

Figure 10 shows the SEM images of strong arc erosion. From the figure, it can be observed that strong arc erosion traces mainly consist of large areas of melting and extensive spattering. The melting traces are flat and cover the wear surface. The individual melting traces are relatively large, with their diameters generally ranging from 30 to 80 μm. This is the result of molten liquid aggregating and solidifying under surface tension. The spattering traces are approximately spherical or strip-shaped, with the individual traces being small and numerous, with diameters of around 3~10 μm. When the amount of spattered molten material is relatively small, it forms an approximately spherical shape during flight due to surface tension, cooling in the air before eventually landing on the wear surface. When a larger amount of molten material is spattered, its flight time in the air is shorter, and it cannot fully cool in the air. When it eventually lands on the wear surface, it forms strip-shaped traces due to the influence of resistance [22,23].

#### 3.2.5. Elemental Changes in Different Worn Surfaces

**Figure 11:**
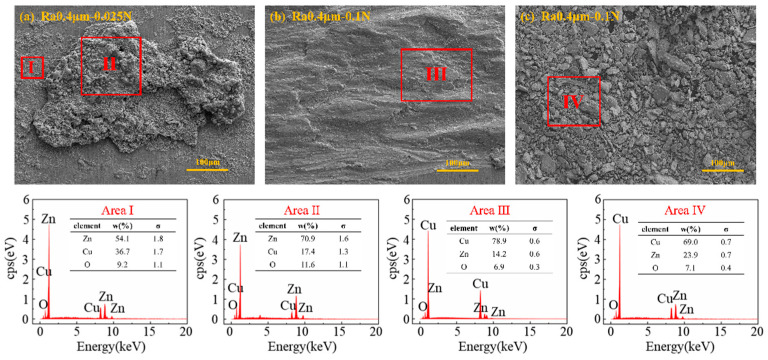
EDS spectra of worn surfaces and wear debris.

Figure 11 shows the EDS spectral characteristics of the worn surfaces and wear debris of the plate specimens under different wear forms. Figure 11a presents the EDS spectrum of the worn surface of the plate specimen under Ra 0.4 μm and 0.025 N, where the damage form is primarily arc erosion. A large amount of the Zn element originating from the wire specimen was detected on the worn surface, with its content being significantly higher than that of Cu, indicating significant material transfer during the arc erosion process. The Zn content in the molten region is slightly higher than in the splashed region. Figure 11b shows the EDS spectrum of the worn surface of the plate specimen under Ra 0.4 μm and 0.1 N, where the wear form is predominantly mechanical wear. The worn surface is mainly composed of Cu, Zn, and O elements, but the Zn content is significantly lower than that detected in the traces caused by arc erosion. Figure 11c presents the EDS spectrum of the wear debris collected under Ra 0.4 μm and 0.1 N, with the components mainly composed of Cu, Zn, and O elements. The presence of O elements was detected in all of the EDS spectra, indicating that oxidation occurred during the friction process.

## 4. Discussion

From the perspective of the current-carrying friction in connectors, it is necessary to study the impact of the initial surface roughness on the performance of a tribological pair. Classical tribology theory divides the entire wear process into running-in, stable wear, and severe wear stages and suggests that after a certain run-in period, the surface roughness of the tribological pair reaches the optimal value, where the wear is minimized. The initial surface roughness of the tribological pair only affects the run-in stage. The mechanical lifespan of the connectors is often only a few hundred cycles (which is very short compared to the wear process described in classical tribology theory) [4], and in the manufacturing process of connectors, the final surface is formed through a “machining + electroplating of precious metals” process (without a run-in period), and the thickness of the precious metal coating is in the micrometer range (excessive run-in is not suitable). Therefore, it is necessary to study the impact of the initial surface roughness of the tribological pair on its performance.

In terms of the distinctiveness of the current-carrying friction, attention must be paid to the service stage of the connector. During the service life of the connector, there is contact resistance heat at its contact surface. When arc erosion occurs, the contact resistance changes significantly (the resistance of the arc follows a nonlinear curve), and a large amount of arc heat causes material melting and splashing, and the newly eroded surface formed affects the subsequent current-carrying friction process. Therefore, it differs from regular dry friction processes, especially after arc erosion occurs, where the change pattern deviates from the typical dry friction behavior.

Under experimental conditions, the lifespan of the tribological pair can be divided into a normal service stage and a failure stage. The contact resistance of the tribological pair performs well during the initial period of the test (mean value around 0.045~0.062 Ω, standard deviation around 0.009~0.017 Ω, with no significant change points); then, it sharply increases until failure (with large standard deviation and significant change points). The variation in the friction coefficient correlates well with the changes in the contact resistance, but failure with severe fluctuations occurs slightly earlier than in contact resistance. Examination of the changes in wear height during a single test revealed that the wear height is relatively low during the normal service stage but increases sharply during the failure stage. During the normal service of the friction pair, the wear surface primarily exhibits furrowing over a large area, with no obvious signs of adhesion, tearing, or arc erosion. As the electrical contact performance deteriorates, the wear surface develops more adhesive and tearing marks, and during the failure stage, a substantial increase in arc erosion is observed (Figure 8).

Under the test conditions, the lifespan of the friction pair increases as the initial surface roughness decreases, with the shortest lifespan being only 0.7 cycles. According to JKR theory, the magnitude of the adhesive force is primarily related to changes in the material and surface energy of the tribological pair (the critical contact depth of indentation) [24,25]. Under the same load, the pressure on the actual contact surface increases as the surface roughness increases. When the surface roughness remains unchanged, the pressure on the actual contact surface increases as the load increases. As the pressure on the actual contact surface increases, the depth of the wire sample pressing into the plate sample increases, resulting in a greater adhesion force, which makes adhesion more likely to occur.

## 5. Conclusions

This study utilized a wire–plate friction pair to investigate the effect of surface roughness on the current-carrying friction performance within the mechanical lifespan of connectors, revealing the influence of the initial surface roughness on their current-carrying friction performance and providing design references for current-carrying friction pairs represented by connectors.

(1) As the surface roughness and load decrease, the contact resistance lifespan increases. Within the range of test parameters, the lifespan of the friction pair reaches a maximum of 320 cycles at a surface roughness of Ra 0.2 μm and a load of 0.025 N, with an average contact resistance of 0.045 Ω and a standard deviation of 0.009 Ω.

(2) During normal service, the primary wear mechanism of the friction pair is furrowing, which microscopically appears as flattening of surface asperities. As the wear mechanism transitions to adhesion, tearing, and plowing, the surface begins to deteriorate, leading to degraded electrical contact performance and failure of the friction pair. Ultimately, the wire specimen melts and breaks due to severe arc erosion.

(3) The impact of arc erosion on the worn surface is significantly greater than that of mechanical wear. Under intense arc erosion, the worn surface exhibits large areas of melting and splashing traces, and the material transfer caused by arc erosion far exceeds that of mechanical wear.

## Data Availability

The original contributions presented in this study are included in the article. Further inquiries can be directed to the corresponding author.

## References

[1] Wang R., Xu L., Zhou Y. (2021). A systematic approach for the reliability evaluation of electric connector. J. Electr. Comput. Eng..

[2] Qian P., Hong L., Chen W., Qian Y., Wang Z., Yao H. (2020). Optimization of the Accelerated Degradation Test Plan for Electrical Connector Contact Pairs Based on a Nonlinear Wiener Process. Math. Probl. Eng..

[3] Braunovic M., Konhits V., Nikolai K., Konchits V.V., Xu L., Lu N., Lin X., Kong Z. (2015). Electrical Contacts: Fundamentals, Applications and Technology.

[4] (2021). General Specification for Super High-Low Temperature Circular Electrical Connector for Space Applications.

[5] Yang F.W. (2023). Prospects for Basic Common Technologies in the Electrical Connector Industry. Electromech. Compon..

[6] Wen S., Huang P., Tian Y. (2018). Principles of Tribology.

[7] Wen B., Pan J., Qian P., Zhang L., Chen W., Zhang J. (2023). Research on the Influence of the Closing Amount of Electrical Connector Contacts on Fretting Wear under a Vibration Environment. Electronics.

[8] Ren W., Wang P., Fu Y., Pan C., Song J. (2015). Effects of temperature on fretting corrosion behaviors of gold-plated copper alloy electrical contacts. Tribol. Int..

[9] Liu X., Cai Z., Liu S., Wu S., Zhu M. (2019). Influence of wear test parameters on the electrical contact performance of brass alloy/copper contactors under fretting wear. J. Mater. Eng. Perform..

[10] Österle W., Dörfel I., Wollschläger N., Gradt T., Wolter C., Reinstädt P., Zeigmeister U., Dmitriev A.I., Nikonov A.Y. (2018). Potential of different nickel coatings for optimizing the sliding behavior of electrical connectors. Tribol. Int..

[11] Ni J., Han L., Pan J., Zheng J., Shi Y., Cui Z., Cai J. (2021). Evolution of contact performance of industry electrical connector based on reliability accelerated testing. Adv. Mech. Eng..

[12] He W., Feng Y., Wu S., Wu K., Ye J., Wang W. (2024). Numerical simulation on the effect of current intensity on electrical contact performance of electrical connectors subject to micro-slip wear. Wear.

[13] Kehong L., Zunqing Z., Zaizhong Z. (2019). Simulation and Experimental Study of the Influence of Temperature Stress on the Intermittent Fault of an Electrical Connector. Exp. Tech..

[14] Sung I., Kim J., Noh H., Jang H. (2016). Effect of displacement and humidity on contact resistance of copper electrical contacts. Tribol. Int..

[15] Guo F., Wang X., Kou J., Li F., Han C. (2022). Impact of Surface Roughness on Pantograph-Catenary Current Collection Quality. IEEE Access.

[16] Du J., Lu M., Fang J., Li W., Chen D. (2024). Current-carrying friction of Ag coatings by additive manufacturing: Uncovering the role of electric current. Mater. Res. Lett..

[17] Wang D., Li F., Huang Q., Wang F., Liu B., Zhao Y. (2024). Effect of normal load on the tribological behavior of graphene coating under current-carrying state. J. Mech. Sci. Technol..

[18] (2020). Geometrical Product Specifications (GPS)—Surface Texture: Areal—Part 1: Indication of Surface Texture.

[19] Yang Z., Song Y., Jiao J., Li W., Shangguan B., Zhang Y. (2024). Optimization of current-carrying friction and wear properties of copper-carbon composite materials based on damage. Tribol. Int..

[20] Zhang Y., Yang Z., Song K., Pang X., Shangguan B. (2013). Triboelectric behaviors of materials under high speeds and large currents. Friction.

[21] Wang P., Zhang H., Yin J., Xiong X., Deng C. (2017). Effects of fiber orientation on wear behavior of copper mesh modified-carbon/carbon composite under electric current. Tribol. Int..

[22] Ren W., Wang T., Zhang X., Zhao M., Wei J. (2018). Research on the rolling and sliding behavior of making contact and associated welding mechanism for low-current switching devices. IEEE Trans. Compon. Packag. Manuf. Technol..

[23] Sun K., Diao D. (2020). Current density effect on current-carrying friction of amorphous carbon film. Carbon.

[24] Zhang Y., Song K., Du S. (2016). Current-Carrying Tribology.

[25] Popov V.L., Li Q., Luo J. (2011). Contact Mechanics and Friction: Physical Principles and Applications.

